# Toward Greener Solvent Systems for the Electrospinning of Cellulose and Its Derivatives

**DOI:** 10.3390/polym18212765

**Published:** 2026-11-06

**Authors:** Hoik Lee

**Affiliations:** 1 Korea Institute of Industrial Technology (KITECH), Ansan 15588, Republic of Korea; hoik@kitech.re.kr; 2 Department of Chemical Engineering, College of Engineering, Chung-Ang University, Seoul 06974, Republic of Korea

**Keywords:** electrospinning, cellulose, cellulose acetate, green solvents, nanofibers, cosolvent, sustainability, ionic liquids

## Abstract

Electrospinning cellulose and its derivatives commonly relies on hazardous solvents, motivating the development of less hazardous alternatives. This narrative review examines conventional and alternative solvent systems, relating polymer solubility, processing conditions, and reported fiber morphology to solvent hazards and practical limitations. Representative studies were identified through targeted searches and reference tracking. For cellulose derivatives, aqueous mixtures of acetone, acetic acid, or ethanol and dimethyl carbonate-based formulations provide alternatives to systems containing hazardous amides or chlorinated solvents. For native cellulose, the reviewed routes include N-methylmorpholine N-oxide, aqueous alkali, ionic liquids, and a switchable solvent system based on 1,8-diazabicyclo[5.4.0]undec-7-ene, carbon dioxide, and dimethyl sulfoxide. Reported mean diameters span 160–1280 nm for cellulose acetate in acetic acid/water and 250–750 nm for cellulose in N-methylmorpholine N-oxide/water. These ranges overlap those obtained with conventional systems, but differences in polymer grade and spinning conditions prevent direct performance comparisons. Lower solvent hazard does not establish lower overall environmental impact: heating, coagulation, washing, carrier polymers, and solvent recovery can introduce additional burdens. The available evidence supports several candidate substitutions, while matched-condition comparisons and quantitative measurements of energy use, recovery yield, waste generation, and productivity are needed to establish their suitability for wider application.

## 1. Introduction

Cellulose, the most abundant natural polymer on earth, is a linear polysaccharide composed of anhydro-D-glucose units linked by β(1→4) glycosidic bonds. Adjacent units are rotated by approximately 180° relative to one another, giving a two-residue structural repeat commonly described as a cellobiose unit [1]. It occurs principally in the cell walls of plants, where it serves a structural role, and is likewise biosynthesized by tunicates, algae, and certain non-pathogenic bacteria [2]. Characteristic of the polysaccharide is an extensive network of intramolecular and intermolecular hydrogen bonds, which confers high crystallinity and mechanical strength while organizing the chains hierarchically into microfibrils and, in turn, macrofibrils [3]. These structural attributes, together with the renewability, biodegradability, biocompatibility, and low cost of the material, have long attracted substantial industrial interest. That interest has grown further as concern over the environmental impact of petroleum-based plastics has increased and attention has turned to bio-based materials [4]. Industrial use of cellulose has historically centered on the pulp and paper sector, but has since broadened, and the polysaccharide together with its derivatives and nanoforms is now processed into membranes, films, coatings, hydrogels, aerogels, fibers, and particles [5,6,7]. Such materials have found application in textiles, food packaging, water remediation, electronics, energy storage, cosmetics, drug delivery, and tissue engineering [8].

Among these forms, fibers produced by electrospinning have attracted particular attention [5,9]. Electrospinning employs an electrostatic field to draw ultrafine fibers from a polymer solution [10]. A high voltage is applied between a solution held at a capillary tip and a grounded collector. When the electrostatic force overcomes the surface tension of the pendant drop, the drop deforms into a conical shape known as the Taylor cone, and a charged jet is ejected from its apex [10]. The jet then undergoes a bending instability that stretches it by several orders of magnitude. In dry electrospinning, solvent evaporation during jet flight solidifies the fibers before collection. In coagulation-based (wet/bath-assisted) electrospinning, a nonsolvent bath is used to remove the solvent and regenerate the polymer. The solidified fibers can be collected as a nonwoven mat, with diameters ranging from tens of nanometers to a few micrometers. The resulting mats offer a high surface area to volume ratio together with high porosity and an interconnected pore structure. Electrospun cellulose fibers have accordingly been examined for affinity membranes, air and water filtration, wound dressings, tissue scaffolds, and drug delivery [11,12,13]. Electrospinning cellulose, however, is not straightforward. Although cellulose hydroxyl groups interact with water, hydration alone does not generally separate the polymer chains. Dissolution requires a solvent to overcome the cohesive interactions between chains and stabilize them in solution. Hydrogen bonding contributes to this cohesion, but hydrophobic association and other nonbonded interactions also contribute to cellulose’s resistance to dissolution in water and most common organic solvents [14]. Thermal degradation also precedes melting, which precludes melt processing [15].

To spin cellulose, therefore, it is either derivatized into an ester or ether such as cellulose acetate (CA), ethyl cellulose (EC), or carboxymethyl cellulose (CMC) and dissolved in water or in an ordinary organic solvent, or dissolved directly in a solvent able to overcome interchain interactions and stabilize the dissolved chains [16]. Where cellulose itself is the target material, the spun derivative is subsequently converted back. CA has been the most widely studied on this basis, since its fibers are readily deacetylated under mild alkaline conditions [17,18]. Either way, the choice of substrate is in effect a choice of solvent.

The solvent is central to every stage of the electrospinning process, from dissolution of the polymer to deposition of the fiber. It determines whether the polymer dissolves at all, and the viscosity, surface tension, and conductivity of the resulting dope are largely set by the solvent system. These properties, together with the applied field, flow rate, and ambient conditions, govern whether a stable jet forms and whether the deposited product is a smooth fiber or a beaded one [19]. In dry electrospinning, the solvent must also evaporate as the jet travels to the collector. A solvent that is too volatile clogs the nozzle before spinning can be sustained. One that is too involatile, on the other hand, leaves the jet still wet when it lands, so that the deposited filaments may fuse or otherwise fail to retain a fibrous form. The window between these two failures is narrow, and where a given solvent falls within it depends on its volatility and on its interaction with the polymer. Solvent selection is therefore decisive in electrospinning, since the system chosen must satisfy all of these requirements at once. Solvents that meet these requirements, however, are for the most part hazardous. CA electrospinning has been established almost entirely on acetone, chloroform, N,N-dimethylformamide (DMF), dichloromethane (DCM), N,N-dimethylacetamide (DMAc), and their mixtures, and native cellulose is more demanding still, calling for N-methylmorpholine N-oxide (NMMO) at elevated temperature, ionic liquids (ILs), or aqueous alkali such as sodium hydroxide (NaOH)/urea. Among these, chloroform and DCM are classified as suspected carcinogens, whereas DMF and DMAc are classified as toxic to reproduction and have drawn regulatory restriction in the European Union [20,21,22]. ILs, often described as green on account of their negligible vapor pressure, vary in this regard; many imidazolium salts have been reported to show aquatic toxicity and to resist biodegradation [23]. Replacing them with less hazardous alternatives, without losing the ability to spin, is therefore an important objective.

Work in this direction has grown considerably in recent years, and a range of solvents has been examined. Water, the most obvious candidate, has been introduced as a cosolvent to moderate the evaporation of volatile conventional solvents. Dimethyl carbonate (DMC) has been used to spin CA. It is attractive on several counts, in that it can be synthesized from carbon dioxide (CO_2_) by some production routes, is of low toxicity, and is readily biodegradable [24]. It has been used together with less volatile cosolvents such as dimethyl sulfoxide (DMSO) or cyclopentanone [25]. γ-Valerolactone (GVL), which can be produced from lignocellulosic biomass, has been employed as a cosolvent for IL systems [26,27]. Previous reviews have discussed polysaccharide electrospinning for regenerative medicine [28], cellulose-based fiber preparation and applications [29,30], and solvent selection in relation to polymer compatibility, toxicological risks, and environmental considerations [31]. Building on this literature, the present review brings together conventional and alternative solvent systems specifically for cellulose and its derivatives, relating reported fiber outcomes to solvent hazards and processing requirements. Differences in substrate properties and experimental conditions are considered when interpreting comparisons across studies.

**Figure 1:**
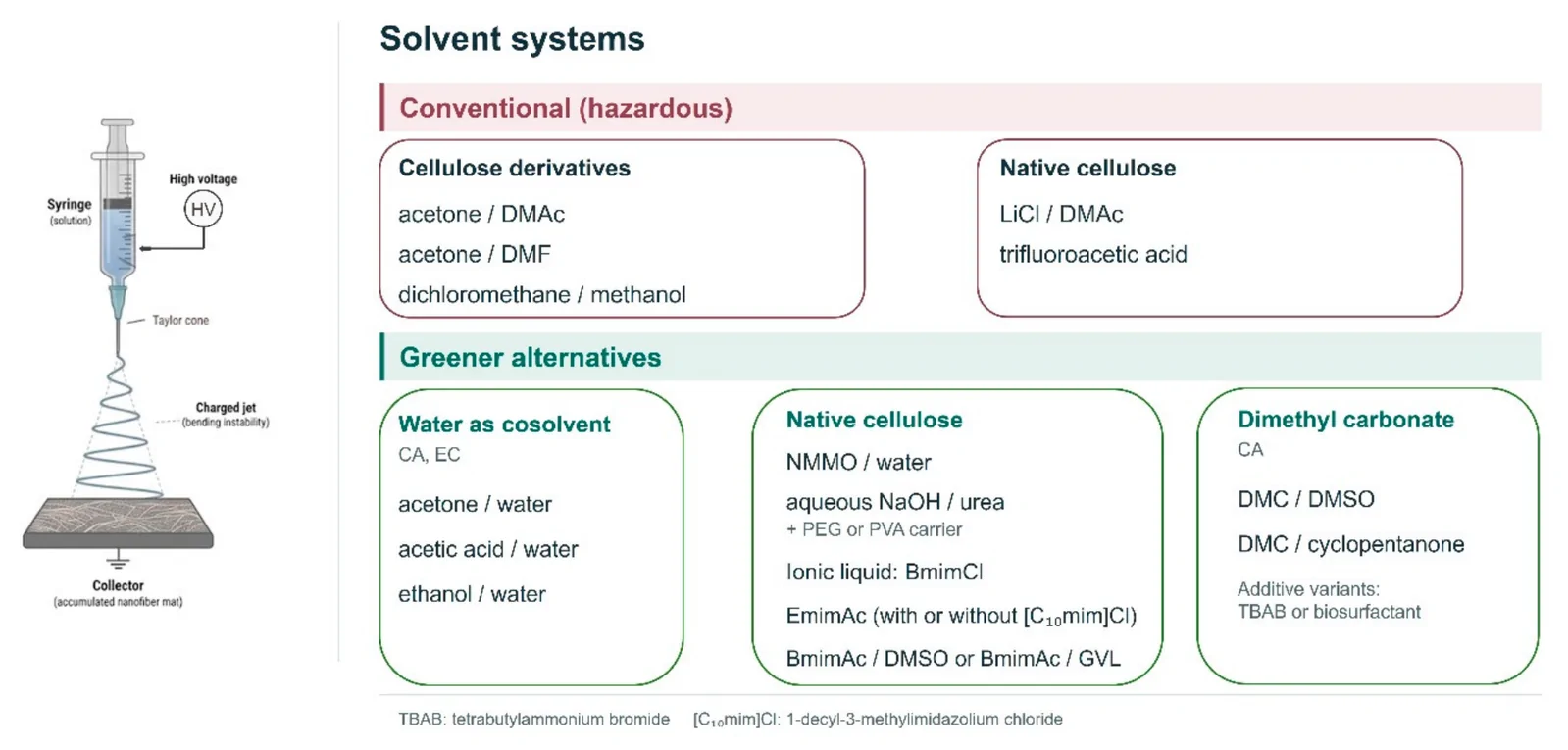
Schematic of the electrospinning of cellulose and its derivatives, and the solvent systems used, grouped into conventional systems and greener alternatives. Abbreviations: NaOH, sodium hydroxide; PEG, poly(ethylene glycol); PVA, poly(vinyl alcohol); BmimCl, 1-butyl-3-methylimidazolium chloride; EmimAc, 1-ethyl-3-methylimidazolium acetate; BmimAc, 1-butyl-3-methylimidazolium acetate.

This review uses greener as a comparative term for solvent systems investigated as alternatives to hazardous conventional formulations. Solvent hazards are evaluated using published Classification, Labelling and Packaging (CLP) classifications and the CHEM21 solvent selection guide; restrictions under Registration, Evaluation, Authorisation and Restriction of Chemicals (REACH) are considered separately [20,21,22,32]. Inclusion as an alternative does not establish that every component is less hazardous or that the complete process has a lower environmental impact. This distinction is particularly relevant to NMMO, ionic liquids, and multicomponent formulations, whose hazards depend on the substance or product used. Processing requirements also affect environmental performance: the coagulation and washing steps used in NMMO and IL systems add water demand, while dissolution and solvent recovery may require heating. Feedstock origin and carrier polymers are additional considerations. Biomass- or carbon dioxide-based production routes are identified where relevant, without assuming that the solvents used in the cited experiments came from those routes. Conventional formulations are presented as practical benchmarks, followed by aqueous systems, specialized solvents for native cellulose, and dimethyl carbonate. Figure 1 maps the solvent systems to the cellulosic substrates. Processing requirements, limitations, and environmental trade-offs are also considered.The aim is to identify feasible substitutions and the evidence still needed to assess them at the process level.

The literature was identified by targeted searches for each solvent system in Google Scholar and on publisher platforms. The searches combined the terms electrospinning or electrospun with cellulose, CA, EC, or regenerated cellulose and with the name of the solvent or solvent class concerned. The search was limited to reports published from 2000 onward, and the last search was carried out in July 2026; the primary studies retained span 2002 to 2026. The reference lists of the retrieved articles were then followed to locate further reports. A study was included when it electrospun cellulose or a cellulose derivative from solution, stated the composition of the solvent system, and reported the morphology or diameter of the product; studies that used electrospraying alone, that did not state the solvent composition, or that employed cellulose only as a dispersed filler in another polymer were not included. Among the greener systems, only those introduced with the explicit aim of replacing or reducing a hazardous solvent are treated. Where several reports described the same solvent system under similar conditions, the earliest and the most fully documented were retained, so that the primary studies cited are representative of each system rather than exhaustive. Hazard classifications were taken from the CLP harmonised classifications, the REACH restriction list, and the CHEM21 solvent selection guide [21,22,32].

## 2. Conventional Solvent Systems

CA predominates among the conventional systems reviewed here and has been spun from a small number of common organic solvents. Acetone, DMAc, DMF, DCM, chloroform, methanol, formic acid, and acetic acid account for the derivative systems summarized here, alone or in binary mixtures. The conditions and outcomes reported for these systems are collected in Table 1.

The first systematic survey was reported by Liu and Hsieh, who electrospun CA (number-average molar mass, Mn, 30,000 g mol^−1^; degree of substitution, DS, 2.45) from acetone, acetic acid, and DMAc and from mixtures of these [33]. None of the three solvents alone supported sustained CA fiber formation under the conditions examined. Acetone and acetic acid gave only a few fibers before the capillary tip became blocked. DMAc gave the opposite outcome, producing beads and no fibers at all across 15 to 25 wt%. Mixtures of acetone and DMAc were more successful, and a 2:1 (*v*/*v*) ratio supported continuous spinning over the widest concentration range, 12.5 to 20 wt%, yielding fibrous membranes with diameters between 100 nm and 1 µm. This system was identified as the most versatile for CA and has been adopted widely since. Deacetylation of the resulting fibers in 0.05 M NaOH/ethanol converted them to cellulose without altering the fiber morphology. Acetone alone was revisited in more detail by Son et al. [34]. Spherical beads formed at 9 wt% and spindle-shaped ones at 13 wt%, and long uniform fibers were obtained only above 17 wt%, the concentration at which the solution viscosity rose steeply as the polymer chains began to entangle. These fibers were relatively coarse, with average diameters of 2.25 and 3.23 µm at 17 and 21 wt% respectively. Even above this critical concentration, spinning could not be sustained, since the rapid evaporation of acetone gelled the solution at the tip and blocked the needle. Majumder et al. also reported concentration-dependent changes in fiber morphology from pure acetone. At 17 wt% CA, cylindrical and flat fibers with reported diameters of 0.8–1.2 µm were obtained, whereas at 19 wt%, flat ribbon-like fibers with transverse dimensions of 50–65 µm were reported [38]. Rapid acetone evaporation can be moderated by adding the less volatile DMAc, and the proportion required was examined by Liu and Tang, who compared acetone-rich (2:1) and DMAc-rich (1:2) mixtures [35]. For the CA grade examined, the critical concentration for continuous fiber formation was about 12.5 wt% in the acetone-rich mixture but about 20 wt% in the DMAc-rich one, so that fibers formed at a lower concentration when acetone predominated. This was attributed to the strong hydrogen bonding between DMAc and CA, which slows the evaporation of DMAc and leaves the jet wet enough to retract into beads; a mixture richer in acetone carries less DMAc and therefore spins at a lower concentration.

Whether other solvents could serve was surveyed more broadly by Tungprapa et al., who screened CA in seven single solvents and three binary mixtures [36]. At a CA concentration of 5% (*w*/*v*), electrospinning solutions prepared with chloroform, DMF, DCM, methanol (MeOH), formic acid, or pyridine produced discrete beads rather than continuous fibers. The acetone solution produced only short, beaded fibers. None of these single-solvent systems therefore yielded a continuous fibrous mat under the conditions examined. Smooth fibers came only from mixed solvents, and from only two of the three tested: 2:1 (*v*/*v*) acetone/DMAc at 12 to 20% (*w*/*v*), with diameters of 0.14 to 0.37 µm, and 4:1 (*v*/*v*) DCM/MeOH at 8 to 12% (*w*/*v*), with 1.10 to 1.58 µm. As with acetone and DMAc, CA in volatile DCM did not form continuous fibers alone, but became spinnable when less volatile MeOH was added. More recently, Sánchez-Cid et al. used a 2:1 acetone/DMF mixture for two cellulose derivatives [37]. CA gave only micrometer-sized particles at 5 wt%, particles together with fibers at 10 and 15 wt%, and continuous fibers 0.31 µm in diameter at 20 wt%. EC, of higher molecular weight, crossed into the fibrous regime at a far lower concentration, giving particles at 2.5 wt% and fibers of 0.19 µm at 5 wt%. At higher concentrations it could not be spun at all, its solutions being too viscous for a stable Taylor cone to form.

**Figure 2:**
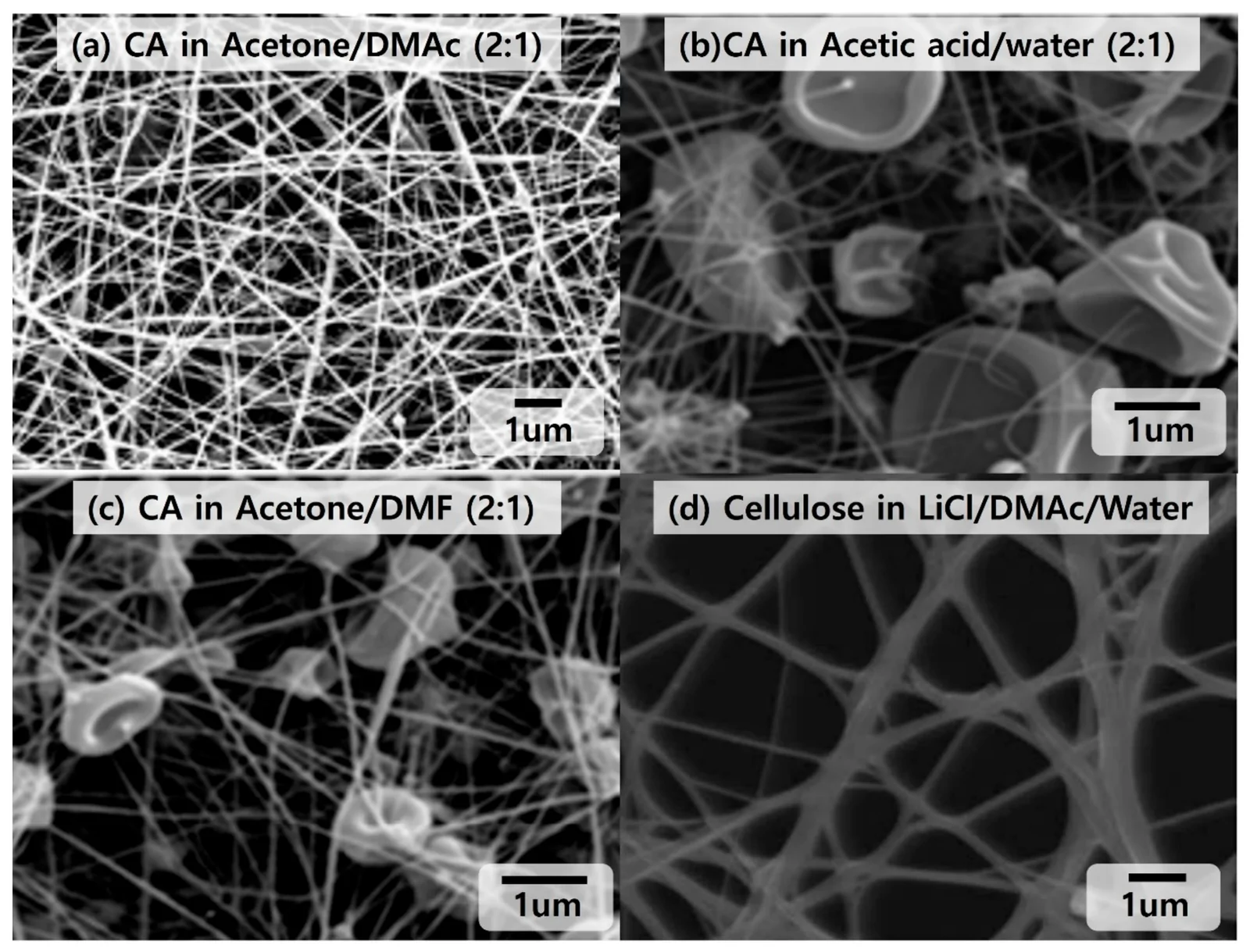
Representative scanning electron micrographs of electrospun CA and cellulose from different solvent systems. The panels are from separate studies with different polymer grades, concentrations, and spinning conditions, so they illustrate the morphologies obtained and are not a direct comparison of the solvents. Polymer concentration and conditions (voltage; distance; flow rate): (**a**) CA from acetone/DMAc (2:1), 12.5 wt%; 12 to 15 kV; 25 cm; no pump; (**b**) CA from acetic acid/water (2:1), 20 wt%; 17 kV; 15 cm; N.R.; (**c**) CA from acetone/DMF (2:1), 15 wt%; 17 kV; 15 cm; N.R.; (**d**) cellulose (DP 1140) from LiCl/DMAc with water coagulation, 3 wt%; 15 to 25 kV; 10 to 20 cm; 0.3 to 1.8 mL/h (ranges for the study). Scale bars: 1 µm. Panel (**a**) adapted with permission from Ref. [33], copyright 2002 Wiley Periodicals, Inc.; (**b**,**c**) reproduced from Ref. [37] (CC BY 4.0); (**d**) adapted with permission from Ref. [39], copyright 2006 Elsevier Ltd. CC BY, Creative Commons Attribution.

Native cellulose is not soluble in such solvents and requires a more aggressive one, conventionally lithium chloride (LiCl) in DMAc. Kim et al. dissolved cellulose in LiCl/DMAc and electrospun it with coagulation in water at the collector, a step needed to remove residual solvent and salt and obtain stable submicron fibers; these measured 250 to 750 nm but were largely amorphous [39]. Trifluoroacetic acid (TFA) dissolves native cellulose directly and, in contrast to LiCl/DMAc, requires no coagulation bath. Ohkawa et al. electrospun cotton and wood pulp cellulose from it at room temperature into fibers of 70 to 130 nm, adding a small amount of DCM to improve spinnability; although the acid partially trifluoroacetylates the cellulose in solution, these ester groups hydrolyze during spinning to leave pure cellulose fibers [40]. Representative fiber morphologies obtained from several of these solvent systems are compared in Figure 2, which shows the range of outcomes, from uniform bead-free fibers to largely particulate deposits, as the solvent is changed.

As described above, cellulose and its derivatives have been electrospun from a variety of solvents, but the systems in common use are mixtures based on DMAc or DMF for the derivatives, while cellulose itself has been spun from LiCl in DMAc or from TFA. These solvents are for the most part hazardous, and several now carry regulatory restrictions. DMF and DMAc are classified as toxic to reproduction and have been restricted under Annex XVII of the REACH Regulation [21,22]; DCM and chloroform, used in some of the derivative systems, are classified as suspected carcinogens; and TFA is a corrosive, environmentally persistent perfluorinated acid [21,41]. These classifications are collected in Table 2. To reduce the reliance on such solvents, greener alternatives have attracted increasing attention in recent years and are now under active development. For reference, the advantages, disadvantages, processing requirements, and main limitations of the greener solvent systems, which are taken up in turn below, are also summarized in Table 3.

**Table 2:** Hazard classifications, CHEM21 rankings, boiling points, and selected REACH Annex XVII restrictions for the solvents, cosolvents, and additives used in the reviewed electrospinning systems.

Substance	CLP Classification ^a^	H-Statements	CHEM21 Ranking ^b^	Boiling Point (°C)/Data Status ^c^	Selected REACH Annex XVII Restrictions ^d^
**Conventional solvents**					
Acetone	Harmonised: Flam. Liq. 2; Eye Irrit. 2; STOT SE 3	H225, H319, H336; EUH066	Recommended	56	—
MeOH	Harmonised: Flam. Liq. 2; Acute Tox. 3 (oral, dermal, inhalation); STOT SE 1	H225, H301, H311, H331, H370	Recommended	65	Entry 69: supply to the general public in windscreen washing or defrosting fluids containing ≥0.6 wt% MeOH
Acetic acid	Harmonised: Flam. Liq. 3; Skin Corr. 1A	H226, H314	Problematic	118	—
Chloroform	Harmonised: Carc. 2; Repr. 2; Acute Tox. 4 (oral); STOT RE 1; Skin Irrit. 2; Eye Irrit. 2	H351, H361d, H302, H372, H315, H319	Highly hazardous	61	Entry 32: restrictions on supply to the general public and dispersive applications at ≥0.1 wt%
Dichloromethane	Harmonised: Carc. 2	H351	Hazardous	40	Entry 59: restrictions on paint strippers containing ≥0.1 wt% dichloromethane
DMF	Harmonised: Repr. 1B; Acute Tox. 4 (dermal, inhalation); Eye Irrit. 2	H360D, H312, H332, H319	Hazardous	153	Entry 76: worker-exposure requirements at ≥0.3 wt%; applicable from 12 December 2023, with sector-specific transitional periods
DMAc	Harmonised: Repr. 1B; Acute Tox. 4 (dermal, inhalation)	H360D, H312, H332	Hazardous	166	Entry 80: worker-exposure requirements at ≥0.3 wt%; applicable from 23 December 2026, or 23 June 2029 for solvent use in man-made fibre production
Formic acid	Harmonised: Flam. Liq. 3; Acute Tox. 4 (oral); Acute Tox. 3 (inhalation); Skin Corr. 1A	H226, H302, H331, H314; EUH071	Problematic	101	—
Pyridine	Harmonised: Flam. Liq. 2; Acute Tox. 4 (oral, dermal, inhalation)	H225, H302, H312, H332	Hazardous	115	—
TFA	Harmonised: Skin Corr. 1A; Acute Tox. 4 (inhalation); Aquatic Chronic 3	H314, H332, H412	Not included ^b^	72	—
**Alternative solvents and cosolvents**					
Water	Not classified as hazardous	—	Recommended	100	—
Ethanol	Harmonised: Flam. Liq. 2	H225	Recommended	78	—
DMC	Harmonised: Flam. Liq. 2	H225	Recommended	90	—
DMSO	Supplier classification: not classified as hazardous	—	Problematic	189	—
Cyclopentanone	Harmonised: Flam. Liq. 3; Skin Irrit. 2; Eye Irrit. 2	H226, H315, H319	Not included ^b^	130–131	—
GVL	Supplier classification: Skin Irrit. 2; Eye Irrit. 2	H315, H319	Problematic	207	—
NMMO, monohydrate	Supplier classification: Repr. 2	H361f	Not included ^b^	N.R.	—
**ILs**					
EmimAc	Supplier classification: Skin Irrit. 2; Skin Sens. 1B	H315, H317	Not included ^b^	N.R.	—
BmimAc	Supplier classification: Skin Irrit. 2; Eye Irrit. 2	H315, H319	Not included ^b^	N.R.	—
BmimCl	Supplier classification: Acute Tox. 3 (oral); Skin Irrit. 2; Eye Irrit. 2; Aquatic Chronic 2	H301, H315, H319, H411	Not included ^b^	N.R.	—
[C_10_mim]Cl (additive)	Supplier classification: Skin Irrit. 2; Eye Irrit. 2	H315, H319	Not included ^b^	N.R.	—
**Other additives and carrier polymers**					
LiCl	Supplier classification: Acute Tox. 4 (oral); Skin Irrit. 2; Eye Irrit. 2	H302, H315, H319	Not included ^b^	1382	—
NaOH	Harmonised: Skin Corr. 1A	H314	Not included ^b^	1390	—
Urea	Supplier classification: not classified as hazardous	—	Not included ^b^	Decomposes before boiling	—
TBAB	Supplier classification: Acute Tox. 4 (oral); Skin Irrit. 2; Eye Irrit. 2; Repr. 2; Aquatic Chronic 3	H302, H315, H319, H361fd, H412	Not included ^b^	N.R.	—
PEG (high molecular weight)	Not classified as hazardous in the cited supplier SDS	—	Not included ^b^	N.R.	Product/form-dependent; applicability of Entry 78 requires assessment
PVA	Not classified as hazardous in the cited supplier SDS	—	Not included ^b^	N.R.	Product/form-dependent; applicability of Entry 78 requires assessment
Sophorolipid biosurfactant (HoneySurf)	Product-specific classification not verified	Not verified	Not included ^b^	Not verified.	Not verified

^a^ Harmonised classifications are those listed in Annex VI of the CLP Regulation; additional supplier classifications may apply. Supplier classifications refer to representative products and may differ with supplier, grade, or formulation. Absence of a harmonized classification does not imply absence of hazards. The entries describe the individual substances or specified products, not the complete electrospinning mixtures. Abbreviations: Flam. Liq., flammable liquid; Acute Tox., acute toxicity; Skin Corr., skin corrosion; Skin Irrit., skin irritation; Eye Irrit., eye irritation; Skin Sens., skin sensitization; Carc., carcinogenicity; Repr., reproductive toxicity; STOT SE/RE, specific target organ toxicity following single/repeated exposure; Aquatic Chronic, long-term aquatic hazard; H-statements, hazard statements; EUH, supplemental European Union hazard statements; TBAB, tetrabutylammonium bromide; [C_10_mim]Cl, 1-decyl-3-methylimidazolium chloride. ^b^ CHEM21 rankings are reproduced fromPrat et al. [32], using the final rankings reported in that publication. “Not included” indicates that the substance or product is absent from these tables. No independent CHEM21 assessment was performed in this review. ^c^ Sources of boiling-point data are provided in Table S1 of the Supporting Information. N.R. = not reported in the cited safety data sheet (SDS); this does not imply that boiling is physically inapplicable. Decomposition before boiling is indicated separately. Not verified = product-specific boiling-point data could not be established. ^d^ Selected REACH Annex XVII restrictions are reported separately from CLP hazard classifications. Applicability depends on the specified use, concentration, material form, and conditions. Entry 59 concerns dichloromethane-containing paint strippers rather than dichloromethane use in general. “—” means that no substance-specific restriction is reported here, not that all uses are unrestricted. Entry 78 concerns synthetic polymer microparticles; its applicability depends on the product and the relevant exclusions or derogations.

**Table 3:** Advantages, disadvantages, processing requirements, and main limitations of the greener solvent systems. Entries summarize the states reported in the studies cited in the text.

System (Substrate)	Advantages	Disadvantages	Processing Requirements	Main Limitations
Water as cosolvent with acetone, acetic acid, or ethanol (CA, EC)	Replaces DMAc or DMF with water; low-hazard solvents (acetone and ethanol Recommended); room-temperature spinning; no coagulation bath; uniform fibers within the working window	Water is a non-solvent, so its content must be held within a narrow range (10–15 wt% in acetone); beading persists at high acetone content; volatile solvent lost by evaporation	Room temperature; conventional dry electrospinning; control of cosolvent ratio	Applies to derivatives only; narrow composition window; polymer precipitates above the water limit
NMMO/water (native cellulose)	Dissolves cellulose directly; established Lyocell solvent with near-complete recovery; fiber crystallinity controllable	Requires heating; thermal degradation of the polymer; energy for solvent reconcentration	Dope held at 70–110 °C; cold-water coagulation bath at the collector; drying; reconcentration required for solvent recovery	Hot processing and the degradation that accompanies it; energy demand of heating and reconcentration
Aqueous alkali, NaOH/urea (native cellulose)	Water-based; no organic solvent; non-derivatizing	Cellulose alone gives particles rather than fibers; carrier polymer needed; alkaline aqueous waste	Precooling to −12 °C for dissolution; fiber-forming carrier (PEG or PVA); washing	Product is a blend rather than pure cellulose under the reported conditions
ILs, imidazolium chloride or acetate (native and bacterial cellulose)	Dissolves cellulose directly; fine fibers; room-temperature spinning with EmimAc; potential solvent recovery	Toxicity and biodegradability depend on the salt; chloride salts need 100 °C; high cost; recovery yield not reported; washing monitored in the EmimAc study	Coagulation bath; washing to remove residual salt; cosolvent (DMSO or GVL) for high-viscosity dopes	Environmental standing depends on the salt chosen; recovery and washing not yet quantified; cost
DMC with cyclopentanone or DMSO (CA)	Low toxicity, biodegradable, CHEM21 Recommended; obtainable from CO_2_ by some production routes; room temperature; no coagulation bath; tunable porosity	Alone gives only coarse, non-uniform microfibers from an unstable jet; cosolvent needed for uniform submicron fibers; salt and biosurfactant additives investigated separately in the cyclopentanone system	Cosolvent ratio controls diameter and porosity; conductivity additives for the cyclopentanone system	Only two reports; early stage; DMC recovery not addressed

## 3. Greener Alternatives

Alternative solvent systems have been investigated to reduce reliance on hazardous conventional formulations [42]. Frey reviewed cellulose and cellulose-derivative electrospinning across several solvent systems [16], whereas Avossa et al. examined benign-solvent definitions and substitution strategies across polymer systems [42]. Here, the emphasis is on how substitution affects fiber formation and processing requirements. Replacing a hazardous cosolvent can preserve conventional dry electrospinning while narrowing the workable composition range. Non-volatile solvents generally introduce coagulation, washing, and recovery steps, and carrier-assisted formulations may yield blends rather than neat cellulose. The systems are organized into aqueous formulations for cellulose derivatives, specialized solvents for native cellulose, and DMC-based formulations for CA. Their reported compositions, polymer concentrations, spinning conditions, fiber diameters, and morphologies are summarized in Table 4. These are candidate alternatives with distinct limitations; their inclusion does not imply a uniform hazard ranking or demonstrated environmental superiority.

### 3.1. Water-Based Solvent Systems

Water is an attractive solvent for electrospinning because of its low toxicity and nonflammability, but its applicability depends on the solubility and spinnability of the target polymer. Native cellulose and commonly used water-insoluble derivatives, including the CA and EC grades discussed here, cannot be dissolved in water alone. Water-soluble derivatives include sodium CMC, hydroxypropyl methylcellulose (HPMC), and methylcellulose (MC), although their solubility and spinnability depend on the polymer grade and solution conditions. For water-insoluble derivatives, water can instead be used as a cosolvent to moderate solvent evaporation and stabilize the jet. CA has been electrospun from acetone in this way [34]. A water content of 10 to 15 wt% in the acetone/water solvent sustained a continuous jet, 5 wt% being insufficient to prevent clogging and 20 wt% rendering the polymer poorly soluble. The addition of water also reduced the heavy beading obtained from acetone alone; hollow, concave beads nonetheless persisted at high acetone contents, where the acetone evaporated too rapidly, at roughly twenty times the rate of water [43]. Acetic acid, a solvent considerably less hazardous than the conventional amides, has likewise been paired with water. CA dissolves in acetic acid above 70 wt%, and a 75:25 acetic acid/water mixture produced uniform, bead-free fibers of about 180 nm at 17 wt% polymer, the concentration below which beads appeared; the fiber diameter could be varied from 160 to 1280 nm by raising the acetic acid content, the coarser fibers resulting from the lower conductivity of the more acidic solutions [44]. Fibers of 180 to 310 nm were obtained from a 3:1 acetic acid/water mixture at 17–19 wt% CA [38], whereas a 2:1 mixture yielded mainly micrometer-scale particles, fibers forming only at the highest concentrations [37]. Ethanol, a widely used solvent in which EC dissolves, has also been paired with water. From an 80:20 ethanol/water mixture, EC formed particles below about 20 wt% and continuous fibers of approximately 3.8 µm at 25 wt%; the water reduced the residual ethanol in the product and was held below 20 vol%, above which the polymer became insoluble [43].

Beyond CA and EC, Frenot et al. investigated CMC, HPMC, and MC using aqueous solvent systems [45]. CMC was electrospun from water with poly(ethylene oxide) (PEO) as a carrier at a 1:1 mass ratio, with selected grades producing fibers of mean diameter 200–250 nm. Subsequent ethanol extraction removed PEO, although retention of the fiber structure depended on the CMC grade. HPMC was electrospun without a carrier polymer from water/ethanol (1:1), yielding mean fiber diameters of 128 and 127 nm for two grades at 2.86 and 2.14 wt%, respectively. MC also formed fibers from this solvent mixture, but these were uneven and partly fused. These results show that aqueous processing can be extended to other cellulose derivatives, although fiber formation and morphology depend on polymer grade, concentration, and drying, with carrier removal adding a further processing step where required.

The solvents used in these systems are all much less hazardous than the amides they replace. Acetone and ethanol are classified as recommended in the CHEM21 guide, and acetic acid, though corrosive, is free of the reproductive toxicity that restricted DMF and DMAc [21,32]. Combined with water in place of the hazardous cosolvent, they avoid the reproductive toxicity associated with DMF and DMAc, although flammability or corrosivity still requires consideration.

### 3.2. Solvents for Native Cellulose

The water-cosolvent systems above apply to the derivatives alone; native cellulose does not dissolve in the solvents they use. Dissolution requires a solvent system that overcomes the cohesive interactions between cellulose chains and stabilizes them in solution [15]. The conventional solvents that do so, LiCl in DMAc and TFA, are hazardous, and greener replacements have been sought among systems that dissolve cellulose directly: NMMO, aqueous alkali, ILs, and the switchable system of 1,8-diazabicyclo[5.4.0]undec-7-ene (DBU), CO_2_, and DMSO.

The first of these is NMMO, the solvent of the commercial Lyocell process. It dissolves cellulose directly and, in that process, is recovered almost completely, so that little of it reaches the environment. Kim et al. drew on this solvent to electrospin cellulose from an NMMO/water solution, the solution being held at 70 to 110 °C during spinning [39]. Since NMMO does not evaporate from the jet in flight, the fibers were coagulated in a cold water bath at the collector, to promote solvent exchange and solidification, followed by vacuum drying at 60 °C. These measured 250 to 750 nm and, unlike the amorphous fibers obtained earlier from LiCl/DMAc, could be given a controlled crystallinity by adjusting the spinning conditions. Intrinsic-viscosity measurements showed the degree of polymerization (DP) decreasing from 1140 to 620 in solution at 100 °C and to 580 in the electrospun fibers; the lower-DP grade changed from 210 to 190 and 180, respectively [39]. Thus, chain shortening had already occurred before fiber collection, with a larger relative loss for the higher-DP grade under the tested conditions. These advantages are bought with energy at two points. The dope must be heated to 70 to 110 °C and held there to keep the cellulose in solution, which brought more extensive thermal degradation of the polymer; and because the solvent is washed into the coagulation bath rather than evaporated, recovering it means removing that water again to reconcentrate the NMMO, which is the energy-demanding step of the recovery loop in the Lyocell process. The near-complete recovery credited to NMMO is therefore a figure from the industrial process; whether it transfers to a laboratory electrospinning line with a coagulation bath is not demonstrated in that study, and neither the energy consumed nor the recovery achieved is reported in the electrospinning study cited here [39]. NMMO thus remains an established and recoverable route to native cellulose fibers of controllable structure, and it replaces the restricted amide and the persistent acid otherwise used for native cellulose; its price is the energy demanded by hot processing and by solvent reconcentration, together with the degradation that accompanies the heating.

Aqueous alkali offers a second route, dissolving cellulose in water without any organic solvent. Fiber formation in electrospinning requires the dissolved chains to entangle enough to be drawn into a continuous jet, and below that threshold the solution breaks into droplets instead [52]. A fiber-forming carrier can assist when the cellulose solution alone does not sustain fiber formation. Qi et al. investigated a 7 wt% NaOH and 12 wt% urea aqueous solution, into which cotton linter cellulose of DP 500 was dissolved after precooling to −12 °C [46]. Under the conditions examined, the cellulose solution alone did not form a stable Taylor cone and produced spherical particles of 100–300 nm rather than fibers. Qi et al. suggested that chain conformation and interchain associations might influence solution surface tension and contribute to this behavior. Continuous fibers appeared only when a fiber-forming polymer was added, either high-molecular-weight poly(ethylene glycol) (PEG) or poly(vinyl alcohol) (PVA); with PEG at about one part to four of cellulose, homogeneous fibers of some 400 nm were formed. The product is then a blend of cellulose with the carrier rather than cellulose alone. Aqueous alkali is thus attractive as a water-based, non-derivatizing solvent, but the fibers obtained in this study contained a carrier polymer.

ILs provide another route and have yielded fine cellulose fibers. These are organic salts of negligible vapor pressure that dissolve cellulose by disrupting its hydrogen bonds. Because they do not evaporate readily, ILs are removed from the fibers using a coagulation bath followed by washing. Recovery from the bath is discussed in the cited studies, but recovery yields and reuse trials are not reported [47,48]. The early work used chloride salts, which are solid at room temperature. Quan et al. spun cellulose from 1-butyl-3-methylimidazolium chloride (BmimCl) with the syringe held in a chamber at 100 °C, obtaining fibers of 500 to 800 nm; these were finest at 4 wt% cellulose, above which the solution grew too viscous and beads formed at the tip [47]. The need for heating was later removed by Freire et al., who used 1-ethyl-3-methylimidazolium acetate (EmimAc), a low-viscosity liquid at room temperature whose acetate anion dissolves cellulose more readily than chloride [48]. At room temperature, an 8 wt% cellulose solution in EmimAc yielded fibers with a mean diameter of 470 nm. Adding 1-decyl-3-methylimidazolium chloride ([C_10_mim]Cl) at an EmimAc/[C_10_mim]Cl mole ratio of 0.90:0.10 lowered the surface tension of the ionic medium and yielded fibers of about 120 nm from an 8 wt% cellulose solution [48]. Acetate salts were used again for bacterial cellulose, of much higher molecular weight, by Vasili et al., whose 1-butyl-3-methylimidazolium acetate (BmimAc) solution could be spun only up to 1 wt% on its own; a cosolvent was therefore added to lower the viscosity of the 3 wt% solution used for spinning [27]. GVL, which can be produced from lignocellulosic biomass and was described by Vasili et al. as a less toxic alternative to DMSO, was used in its place to reduce solution viscosity and enable electrospinning [27].

Thermal stability after regeneration must be distinguished from preservation of chain length during dissolution. Quan et al. attributed the slightly lower thermal stability of BmimCl-derived fibers than regenerated films partly to degradation during spinning [47]. For fibers prepared at room temperature in EmimAc-based media, Freire et al. reported thermal degradation at temperatures about 10 K lower than for raw cellulose [48]. These thermogravimetric observations do not quantify molecular-weight or DP loss, because regeneration also changes crystalline structure. Chain shortening may reduce regenerated-fiber strength, but paired molecular-weight/DP and tensile measurements are needed to separate this contribution from crystallinity and orientation; this relationship was not quantified in the cited NMMO and IL electrospinning studies [39,47,48].

ILs are often called green because they do not evaporate, but low vapor pressure limits evaporative solvent loss without establishing low toxicity and is not the basis on which they are counted as greener here. That basis is the one set out in the Introduction, the hazard of the solvent itself, and on it the class divides. The aquatic toxicity and biodegradability of imidazolium salts depend strongly on the cation alkyl chain length and the anion; the longer-chain members are toxic to aquatic organisms and resist biodegradation, whereas EmimAc has been described as having low toxicity and favorable biodegradability [48]. This provides a reported rationale for its selection as an alternative solvent, although the environmental profile must be considered separately for each IL. Potential recyclability does not establish recovery efficiency or environmental benefit. The cited studies describe transfer of ILs into the coagulation bath and subsequent washing liquids, but do not report quantitative solvent losses or wastewater-treatment performance [27,47,48]. Freire et al. repeatedly washed the fibers with water and ethanol until no IL was detected in the wash solvent by proton and carbon-13 nuclear magnetic resonance spectroscopy; infrared spectroscopy and elemental analysis also supported removal from the fibers [48]. These results do not provide a quantitative residual-IL concentration in the fibers, and incomplete removal can cause plasticization and fiber fusion [48]. The available studies do not provide a common basis for comparing residual IL levels, recovery yields, solvent losses, or wastewater-treatment performance across systems. These findings support the consideration of selected ILs, particularly EmimAc, as potentially greener alternatives for cellulose electrospinning, combining a reported favorable hazard profile with fine-fiber formation at room temperature. Their overall environmental benefit nevertheless depends on solvent recovery and the water and energy required for washing, which remain insufficiently quantified.

A further route uses the DBU/CO_2_/DMSO switchable solvent system. Heidari et al. dissolved cellulose in this system under CO_2_ and investigated the electrospinning of microcrystalline cellulose and cellulose pulp [51]. Microcrystalline cellulose predominantly yielded particles, even at 10 wt%, whereas 4 wt% cellulose pulp produced bead-free standing fibers with a mean diameter of approximately 500 nm. The study also investigated PVA and PEO as carrier polymers, distinguishing these blended systems from the direct spinning of cellulose pulp without a carrier. The contrasting outcomes for the two cellulose feedstocks show that successful dissolution alone does not ensure fiber formation and that the choice of cellulose remains important to spinnability. This route broadens the solvent options for direct cellulose electrospinning, while its overall environmental performance also depends on the hazards of DBU and DMSO, solvent recovery, and processing energy.

### 3.3. Dimethyl Carbonate

A more recent effort has sought to replace the conventional organic solvents for CA with DMC. The solvent is of low toxicity and biodegradable and can be made from carbon dioxide and MeOH [24], and it is classified as recommended in the CHEM21 guide [32]. It is made industrially by several routes: oxidative carbonylation of MeOH with carbon monoxide, transesterification of cyclic carbonates that are themselves obtained from carbon dioxide and an epoxide, and, still at a developing stage, direct synthesis from carbon dioxide and MeOH [24].

Like other volatile solvents, it does not give uniform submicron fibers on its own: its rapid evaporation and negligible conductivity leave the jet unstable, so that only coarse, non-uniform fibers of several micrometers are obtained, and a less volatile cosolvent is added to stabilize the jet. Changing the solvent ratio affects both CA solvation and jet drying: preferential evaporation of DMC is expected to leave a cosolvent-rich liquid phase during solidification, helping explain the composition-dependent fiber diameter and surface morphology [25,49].

The cosolvent has been either cyclopentanone or DMSO. Both DMC and cyclopentanone dissolved CA, but each alone gave irregular fibers at 15% (*w*/*v*), motivating the use of mixtures [25]. In the DMC/cyclopentanone system, DMC can be produced through CO_2_-based routes [24], while cyclopentanone can be obtained by hydrogenation of biomass-derived furfural [53]. The mixture had low conductivity, so tetrabutylammonium bromide (TBAB) was added at 1–10 wt% relative to the CA mass. A loading of 2.5 wt% yielded uniform fibers from 12.5% (*w*/*v*) CA in DMC/cyclopentanone at 75:25 (*v*/*v*). A sophorolipid biosurfactant was investigated separately to improve fiber uniformity [25]. In the solvent-ratio series without additives, 15% (*w*/*v*) CA gave porous fiber surfaces in DMC-rich mixtures, whereas cyclopentanone contents of 50 vol% or more produced smooth surfaces; the authors linked this difference to faster DMC evaporation. Thermal-profile differences were attributed partly to possible residual solvent, but this interpretation did not quantify the amounts of DMC and cyclopentanone retained [25].

For 18 wt% CA, the solution changed from slightly cloudy in pure DMC to transparent in DMC/DMSO mixtures, consistent with improved solubilization [49]. DMSO, being far more polar, raised the conductivity on its own and required no additives. From pure DMC the jet was unstable and the product consisted of non-uniform, porous microfibers of mean diameter 2.9 µm that adhered to the collector. As the proportion of DMSO was increased, the solution became less viscous and more conductive and the fibers finer, smoother, and narrower in their distribution, their mean diameter falling to 0.5 µm at a 50:50 (*w*/*w*) ratio. Across the solvent-ratio series, crystallinity ranged from about 7 to 19%, and tensile strength reached about 7 MPa. The spectroscopic signal of residual DMSO in freshly collected fibers was more pronounced at DMC/DMSO ratios of 67:33 and 50:50 (*w*/*w*), and disappeared after heating at 120 °C for 24 h [49]. Thus, smooth fiber morphology did not establish complete solvent removal during jet flight.

The role of DMSO should therefore be considered in terms of both solvent hazard and processing requirements. Although DMSO can replace hazardous amide solvents, it is classified as Problematic in the CHEM21 guide, whereas DMC is Recommended; the presence of DMSO alone does not establish the environmental performance of the mixture [32]. A DMC-free alternative was demonstrated by Neukirch et al., who electrospun CA using acetone/DMSO (77.5:22.5, *v*/*v*) as a replacement for acetone/DMF [50]. This system also required a balance between jet stability and solvent evaporation: lower DMSO contents caused nozzle clogging, whereas a higher content produced wet deposits. Thus, replacing DMC with acetone provides another route to CA fibers, but solvent composition remains critical to stable spinning and adequate drying.

### 3.4. Polymer–Solvent Interactions and Cross-System Comparison

The preceding studies show that polymer–solvent compatibility and electrospinnability must be assessed separately. For CA, Hansen solubility parameters (HSPs) resolve cohesive interactions into dispersion, polar, and hydrogen-bonding contributions. The distance between a solvent and the polymer in this three-dimensional space can be normalized by the polymer interaction radius to give the relative energy difference (RED). A value below unity conventionally indicates affinity within the adopted solubility sphere [25,38]. Majumder et al. used Hansen parameters and a Teas diagram to interpret the different behavior of acetone, acetone/DMAc, and acetic acid/water [38]. This approach provides a basis for screening compositions, but a favorable location in the diagram does not establish continuous fiber formation.

Reported HSP analyses also help explain the roles of cosolvents in DMC formulations. Oldal et al. reported RED values of 1.37 for DMC and 0.76 for cyclopentanone relative to the CA parameter set used in their study, indicating greater predicted affinity for cyclopentanone [25]. Castro et al. found that adding DMSO shifted the calculated mixture coordinates further inside the CA solubility sphere; this interpretation was supported by the change from a slightly cloudy solution in pure DMC to transparent mixed-solvent solutions [49]. These observations support improved solvation as one contribution to the changes in solution behavior. However, the CA parameter sets adopted by Majumder et al. and Oldal et al. differ [25,38], so their numerical distances and interaction radii should be interpreted within each study rather than combined into a universal solvent ranking.

Thermodynamic compatibility concerns the free-energy balance of mixing, whereas the rate and extent of dissolution also depend on access to the polymer chains and disruption of ordered regions. In polymer-solution models, the Flory–Huggins interaction parameter, χ, describes solvent quality, with lower values generally indicating more favorable mixing under comparable conditions. Liu and Hsieh used this framework to discuss CA dissolution and considered DMAc more thermodynamically favorable than acetic acid or acetone [33]. Their CA–DMAc interaction parameter was assumed from structural considerations, however, rather than measured. Moreover, DMAc alone produced beads in their electrospinning experiments, while acetone/DMAc mixtures supported fibers. This example demonstrates why a proposed thermodynamic preference should not be treated as an experimentally established ranking of electrospinning performance.

Hydrogen-bonding characteristics further distinguish CA from native cellulose. Acetyl substitution changes the balance of hydroxyl donors and ester acceptor groups, so the interaction requirements of CA differ from those of the unmodified polymer. For CA, Liu and Tang attributed the persistence of wet jets in DMAc-rich solutions partly to strong CA–DMAc hydrogen bonding, linking favorable polymer–solvent interactions to delayed solvent removal [35]. For native cellulose, the solvent must stabilize separated chains against reassociation. In cellulose-dissolving ILs, hydrogen-bond acceptance by chloride or acetate anions helps compete with cellulose–cellulose hydrogen bonds [47,48]. Vasili et al. likewise related the dissolution of bacterial cellulose in BmimAc to acetate–hydroxyl interactions [27]. Thus, the relevant issue is the type and balance of interactions; a large hydrogen-bonding contribution alone does not establish compatibility. CA solubility-sphere parameters cannot be transferred directly to native cellulose in NMMO or IL formulations.

Dissolution must then be connected to chain association, entanglement, and solidification. In the NaOH/urea system, Qi et al. obtained particles from dissolved cellulose alone and fibers after adding a carrier polymer; they associated the change with altered intermolecular interactions and chain flexibility [46]. This separates successful dissolution from the viscoelastic response needed to resist jet breakup. HSPs and thermodynamic arguments should therefore be interpreted together with polymer molecular weight, degree of substitution, concentration, and the route of solvent removal. The studies discussed above do not provide a common experimentally validated set of HSPs or interaction parameters for all formulations. Their reported values support interpretation within individual systems, while quantitative prediction across CA, native cellulose, and ionic or aqueous multicomponent solvents remains limited.

These interaction-based considerations must be combined with the processing requirements of each solvent system. Solution viscosity, surface tension, electrical conductivity, and solvent evaporation influence jet stability and, consequently, bead formation and fiber diameter [33,48,49]. Solvent composition must then balance jet drying against premature evaporation. For CA, adding a less volatile cosolvent can suppress nozzle clogging, whereas excessive retention of solvent can produce wet or fused deposits [33,34,35,50]. Water addition is similarly limited by substrate solubility: it can stabilize spinning within a suitable composition range but cause poor dissolution at higher fractions [34,43]. Conductivity provides another means of adjusting fiber formation. In the DMC/DMSO system, 18 wt% CA solutions showed higher conductivity, lower viscosity, and finer, more uniform fibers as the DMSO fraction increased; a 50:50 (*w*/*w*) solvent mixture yielded smooth, bead-free fibers with a mean diameter of approximately 0.5 µm [49]. Because these properties changed together, the diameter reduction cannot be assigned to conductivity alone. The solvent systems are therefore assessed here in terms of demonstrated fiber formation under the reported conditions; these observations do not establish independently optimized parameters or equivalent performance across systems.

Individual studies also reported changes in surface porosity, crystallinity, and tensile strength with solvent composition. In the DMC/cyclopentanone system, surface porosity increased with the DMC fraction [25]. For 18 wt% CA in DMC/DMSO, mat tensile strengths were 2.23 ± 0.50 and 7.21 ± 1.00 MPa at solvent ratios of 83:17 and 50:50 (*w*/*w*), respectively [49]. Pore-size distributions derived from scanning electron microscopy for 3 wt% bacterial cellulose meshes showed fewer larger pores with BmimAc/DMSO than BmimAc/GVL [27]. BmimAc/DMSO meshes also absorbed more water and phosphate-buffered saline than pristine bacterial cellulose [27]. CA meshes from acetone/DMSO (77.5:22.5, *v*/*v*) showed mean water contact angles of approximately 53–63° at 12–16% (*w*/*v*) CA [50]. Fiber-surface pores differ from inter-fiber pores, and absorption does not measure wettability or filtration performance. Comparable datasets covering all these properties, including filtration efficiency or permeance, are lacking. These results do not establish equivalent membrane performance across solvent systems.

To interpret Table 1 and Table 4, comparisons first distinguish native cellulose, its derivatives, and carrier-containing formulations. Polymer grade (molecular weight or DP and, where applicable, DS), concentration, additives, and processing conditions, including solvent removal, are then considered. Within-study solvent-composition changes provide the clearest basis for discussing solvent-related trends when these factors are held constant. If they vary or are not reported, differences in fiber morphology or diameter are treated as combined formulation and processing outcomes. Across studies, the results document fiber formation under the reported conditions; they do not isolate solvent effects or support a solvent-performance ranking.

**Figure 3:**
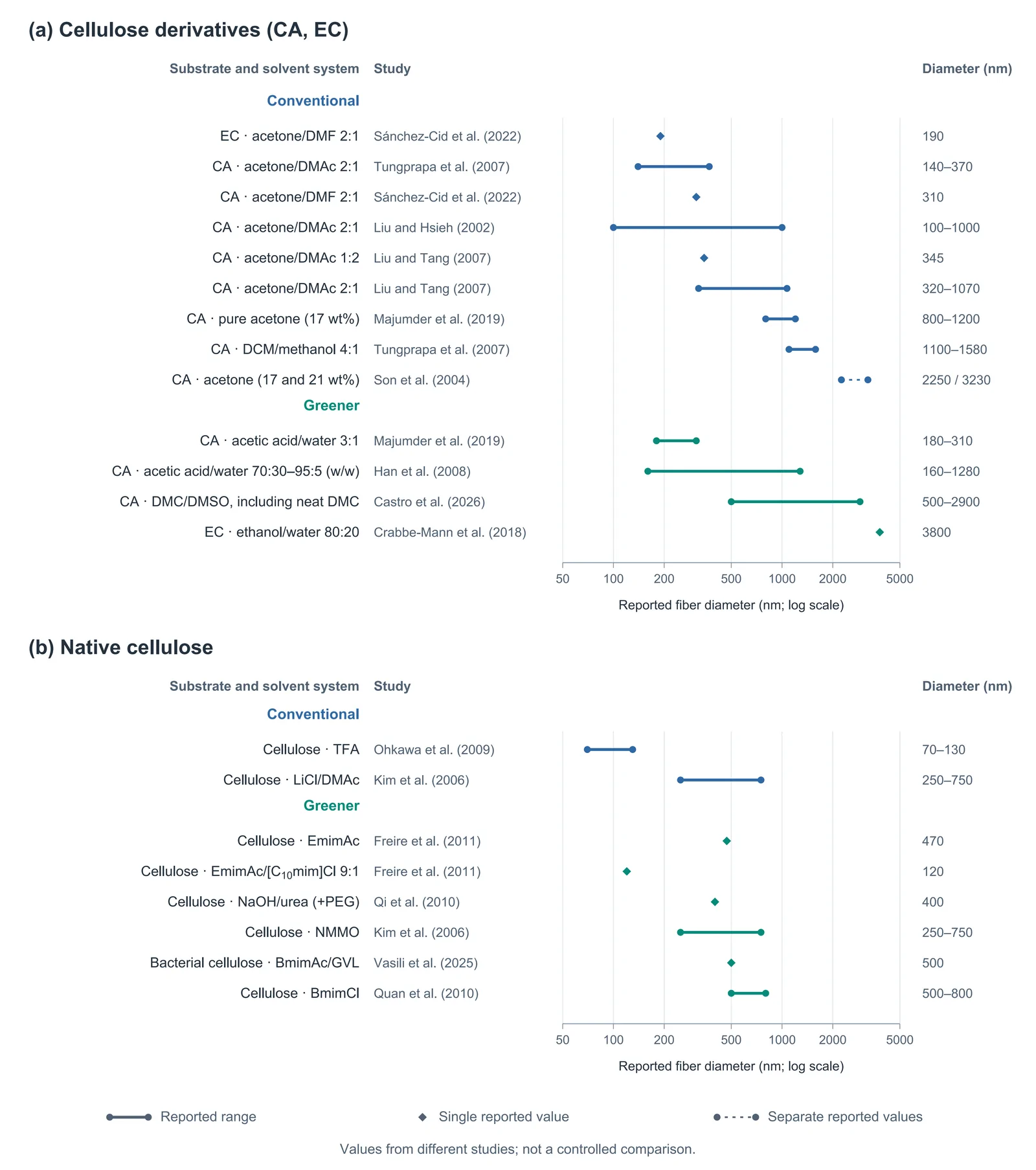
Reported electrospun fiber diameters, grouped by substrate and solvent system: (**a**) cellulose derivatives (CA and EC) and (**b**) native cellulose. Solid bars span the reported diameter values summarized for each entry; diamonds indicate individual representative values, and circles joined by a dotted line indicate two separate reported values (CA from acetone at 17 and 21 wt%). Neat EmimAc and EmimAc/[C_10_mim]Cl (9:1 mol/mol) are shown separately. The studies used different polymer grades, concentrations, and spinning conditions; the figure is therefore a survey of reported outcomes, not a controlled comparison of solvent performance. Selected systems are shown; additional formulations and outcomes are summarized in Table 4. Data sources (identified by author and year in the figure): Vasili et al. (2025) [27]; Liu and Hsieh (2002) [33]; Son et al. (2004) [34]; Liu and Tang (2007) [35]; Tungprapa et al. (2007) [36]; Sánchez-Cid et al. (2022) [37]; Majumder et al. (2019) [38]; Kim et al. (2006) [39]; Ohkawa et al. (2009) [40]; Crabbe-Mann et al. (2018) [43]; Han et al. (2008) [44]; Qi et al. (2010) [46]; Quan et al. (2010) [47]; Freire et al. (2011) [48]; Castro et al. (2026) [49].

The reported fiber diameters of the conventional and greener systems are collected in Figure 3, separated into the cellulose derivatives (Figure 3a) and native cellulose (Figure 3b). Within each substrate class the greener systems span much the same range of reported diameters as the conventional ones. Because the studies still differ in molecular weight, concentration, and spinning conditions, this overlap shows that the greener systems reach the same diameter regime; it does not establish that a given greener system would match a given conventional one under the same conditions, which only a controlled comparison could show.

The process-level characteristics of these systems, namely solvent toxicity, processing temperature, energy demand, solvent recovery, water use, and waste generation, are compared in Table 5.

**Table 5:** Critical comparison of the greener solvent systems, with a conventional benchmark, across process-level dimensions. Entries reflect the states reported in the cited studies and the hazard classifications used in Table 2; water use and waste entries follow the process features (coagulation bath, washing, additives) described in those studies. The primary studies rarely report quantitative metrics, so the comparison is qualitative. Energy-demand descriptors refer to reported heating or cooling requirements, not measured total energy consumption. “High” and “low” indicate reported process requirements rather than measured quantities; these descriptors do not establish environmental impacts per unit mass of fibers.

System (Substrate)	Solvent Hazards	Processing Temp.	Energy Demand	Solvent Recovery	Water Use	Waste Generation
Conventional benchmark: acetone/DMAc (CA); LiCl/DMAc (cellulose)	DMAc Repr. 1B, Hazardous, REACH-restricted [21,22,32]	Room-temperature spinning; heated collector for LiCl/ DMAc	No heating specified for the CA benchmark; collector heating and subsequent drying for LiCl/DMAc; total energy not quantified	Not recovered (evaporates); LiCl/DMAc to coagulation bath	Low (CA); high (cellulose, coagulation bath) [39]	Amide-containing vapor and bath waste
Water as cosolvent: acetone/, acetic acid/, ethanol/water (CA, EC)	Low; acetone and ethanol Recommended, acetic acid Problematic (corrosive), water none [21,32]	Room temp.	Not quantified; room-temperature spinning without a coagulation bath	Volatile component lost by evaporation; not recovered	Low; water a minor cosolvent, no coagulation bath	Volatile-organic emissions; no coagulation-bath waste
NMMO/water (cellulose)	NMMO monohydrate: suspected of damaging fertility (supplier classification); not included in the cited CHEM21 tables.	70–110 °C [39]	High (heated dope; solvent reconcentration after coagulation)	Near-complete recovery refers to the industrial Lyocell process [39].	High (coagulation bath)	Low solvent waste if recovered; thermal degradation of the polymer
Aqueous alkali, NaOH/urea + carrier polymer (cellulose)	No organic solvent; NaOH corrosive, urea low-hazard	Precooling to −12 °C [46]	Not quantified; precooling required for dissolution	Not recovered; alkali in wash water	High (aqueous solvent and washing)	Alkaline aqueous waste; reported product is a cellulose/carrier blend [46]
ILs, imidazolium chloride or acetate (±GVL) (cellulose, bacterial cellulose)	Structure-dependent; longer-chain imidazolium toxic and persistent [23]; EmimAc described as having low toxicity and favourable biodegradability in Ref. [48].	100 °C (chloride) [47] to room temp. (acetate) [48]	Heating required for BmimCl; room-temperature spinning demonstrated with EmimAc. Overall energy consumption not quantified [47,48].	Recovery from the coagulation bath is discussed; recovery yield and reuse trials are not reported [47,48].	High (coagulation bath)	Coagulation water carries residual salt; fibers require washing; recovery reduces loss, cost remains high
DMC + cosolvent (cyclopentanone or DMSO) (CA)	DMC: Recommended; DMSO: Problematic (CHEM21 [32]). Cyclopentanone: flammable liquid; skin and eye irritant	Room temp.	Not quantified; room-temperature spinning, with additional heating at 120 °C for residual DMSO removal in the DMC/DMSO system [49].	Volatile DMC lost by evaporation; recovery not addressed	Low; no coagulation bath	Additives (salt or biosurfactant) in the cyclopentanone system [25]; residual-solvent removal required for DMC/DMSO [49]; waste quantities not reported

Table 5 shows trade-offs rather than a single preferred solvent system. Aqueous and DMC-based formulations for cellulose derivatives permit room-temperature spinning without a coagulation bath, but these features alone do not establish low total energy demand. The DMC/DMSO study, for example, required heating at 120 °C to remove residual DMSO. Recovery of the volatile solvents was not demonstrated. For native cellulose, NMMO and ILs offer potential recovery routes but require coagulation and washing; heating is also required for NMMO and chloride-based ILs. The aqueous alkali formulation avoids organic solvents but yields a cellulose/carrier blend. Substitution can reduce selected hazards while introducing additional demands for energy, water, solvent separation, or carrier removal. The balance depends on the substrate, formulation, and intended application. Quantitative comparisons remain limited by the lack of measurements of energy use, solvent-recovery yield, and waste generation on a common basis.

Evidence for industrial operation remains limited. Qi et al. reported approximately 2 h of continuous spinning of cellulose/PEG in NaOH/urea, yielding a coarse, inhomogeneous mat [46]. Freire et al. described IL recovery by evaporating water from the coagulation bath, without reporting recovery yields or reuse trials [48]. Castro et al. cited a lower purchase price for DMSO than cyclopentanone in 2025, but did not evaluate total electrospinning costs [49]. These observations do not establish sustained productivity or economic feasibility. Comparable data on collected-fiber output, long-term operation, equipment corrosion, and operating costs are still needed to assess industrial scalability.

Detailed quantitative environmental assessment remains an important subject for a dedicated future review. Such work would require more complete and comparable process data, together with justified assessment criteria and process boundaries, to complement the present review’s focus on solvent selection and reported electrospinning feasibility.

## 4. Conclusions and Future Outlook

The electrospinning of cellulose and its derivatives has for the most part been carried out with hazardous solvents: the derivatives from mixtures containing DMAc, DMF, or DCM, and native cellulose from LiCl in DMAc or from TFA. Several of these solvents are now restricted under REACH, which has driven the search for greener alternatives. This review brings together aqueous and mixed-solvent routes for cellulose derivatives, DMC-based systems for CA, and alternative routes for native cellulose using NMMO, aqueous alkali, ILs, and the DBU/CO_2_/DMSO switchable solvent system.

The reported diameter ranges of the greener and conventional solvent systems overlap (Figure 3), demonstrating that alternative solvents can support fiber formation within diameter regimes already achieved using conventional solvents. This provides a practical basis for identifying candidate solvent substitutions, while equivalence in fiber quality and process performance remains to be established under matched conditions.

For the CA and EC mixed-solvent systems discussed here, solvent composition balances polymer solubility, jet stability, and evaporation. Water can replace a hazardous organic cosolvent within a suitable composition range, while DMC provides an alternative volatile solvent for CA. Water-soluble derivatives offer additional routes: CMC can be electrospun from water with a PEO carrier, whereas HPMC and MC have been processed from water/ethanol mixtures without a carrier. These systems broaden the available solvent options, although polymer grade, drying behavior, and carrier removal where required remain important practical considerations. Native cellulose stands apart. Dissolving its chains requires specialized solvent systems, and subsequent solvent removal can complicate spinning: NMMO and the ILs are non-volatile, cannot leave the jet in flight, and require a coagulation bath at the collector, while the aqueous alkali formulation reviewed here required a fiber-forming carrier.

Each alternative retains practical limitations. For CA, the acceptable water fraction depends on the organic solvent and polymer grade; excessive water can reduce solubility and interrupt spinning. NMMO requires hot processing and solvent reconcentration, and thermal degradation must be controlled. Its monohydrate also carries a supplier classification for suspected reproductive toxicity (Table 2), so it should not be described as intrinsically benign. IL hazards depend on the cation and anion, and negligible vapor pressure does not establish low aquatic toxicity or biodegradability [23]. Recovery yields and washing requirements remain insufficiently quantified. The reviewed aqueous alkali formulation yields a cellulose/carrier blend, while the DMC systems require cosolvents whose hazards and drying requirements must be included in the assessment. DMC is recommended in the CHEM21 guide [32] and can be produced through carbon dioxide-based routes [24], but neither property establishes the environmental performance of the complete spinning process. Robustness and scalability therefore require evaluation against conventional formulations under comparable conditions.

Deep eutectic solvents (DESs) are also discussed as potential greener media for cellulose processing. However, their practical use in direct cellulose electrospinning remains insufficiently established: cellulose compatibility or dissolution alone does not ensure the rheology and solidification needed for continuous fiber formation, and results obtained for other polysaccharides cannot be transferred directly to cellulose [54,55,56].

Four priorities follow from these limitations. First, carbonate-based formulations should be examined systematically to establish how solvent identity, cosolvent identity, and composition affect dissolution, jet stability, drying, and fiber morphology. Second, direct spinning of native cellulose requires further work on solvent hazards, polymer degradation, and carrier-free fiber formation, including evaluation of lower-toxicity IL candidates and aqueous alkali formulations. Third, comparisons should use matched polymer grades and a common experimental framework. Polymer concentration and spinning conditions should be controlled where feasible, with any solvent-specific optimization reported explicitly. Fiber diameter, uniformity, yield, and residual solvent should be measured alongside energy use, solvent-recovery yield, water consumption, and waste on a common functional basis, such as a unit mass of dry fibers. Fourth, scale-up studies should report productivity, dope stability during extended operation, solvent emissions, recovery performance, and cost. These measurements would help distinguish formulations that merely demonstrate fiber formation from those suitable for sustained production. The available studies establish feasible alternatives; broader adoption depends on showing that their processing and environmental advantages persist beyond laboratory demonstrations.

## Figures and Tables

**Table 1:** Conventional solvent systems for electrospinning of cellulose and its derivatives.

Substrate	Solvent System	Conc. ^a^	Conditions ^b^ (kV; cm; mL/h)	Fiber Diameter	Morphology	Ref
CA (M_n_ 3.0 × 10^4^, DS 2.45)	acetone	15, 20 wt%	5–10; 18; N.R.	—	few fibers, tip clogged	[33]
CA	acetic acid	13 wt%	10–15; 18–20; N.R.	—	few fibers	[33]
CA	DMAc	15–25 wt%	10–15; 15–25; N.R.	—	beads only	[33]
CA	acetone/DMAc 2:1	12.5–20 wt%	10–15; 15–25; N.R.	0.1–1 µm	smooth	[33]
CA (M_n_ 3.0 × 10^4^, DS 2.45)	acetone	9, 13 wt%	12; 8; 10	—	spherical/spindle beads	[34]
CA	acetone	17, 21 wt%	12; 8; 10	2.25, 3.23 µm	coarse fibers; tip clogs	[34]
CA (M_w_ 3.5 × 10^4^)	acetone/DMAc 2:1 (*v*/*v*)	20, 25 wt%	8; 15; N.R.	0.32–1.07 µm	smooth	[35]
CA	acetone/DMAc 1:2 (*v*/*v*)	25 wt%	8; 15; N.R.	0.345 µm	smooth	[35]
CA (M_w_ 3.0 × 10^4^, DS 2.4)	acetone, CHCl_3_, DMF, DCM, MeOH, formic acid, pyridine (each single)	5% (*w*/*v*)	15; 15; N.R.	—	discrete beads (acetone: short beaded)	[36]
CA	acetone/DMAc 2:1 (*v*/*v*)	12–20% (*w*/*v*)	12; 15; N.R.	0.14–0.37 µm	smooth (14–20% (*w*/*v*))	[36]
CA	DCM/MeOH 4:1 (*v*/*v*)	8–12% (*w*/*v*)	15; 15; N.R.	1.10–1.58 µm	smooth	[36]
CA (M_w_ 3.0 × 10^4^)	acetone/DMF 2:1	5, 10, 15 wt%	17; 15; N.R.	0.15–0.16 µm	particles (5 wt%) → particles + fibers (10–15 wt%)	[37]
CA	acetone/DMF 2:1	20 wt%	17; 15; N.R.	0.31 µm	fibers	[37]
EC (M_w_ 4.5 × 10^4^)	acetone/DMF 2:1	2.5, 5 wt%	17; 15; N.R.	0.19 µm (5 wt%)	particles → fibers	[37]
CA (M_n_ 3.0 × 10^4^)	pure acetone	17 wt%	20; 10; 1.5	0.8–1.2 µm	cylindrical and flat fibers; no beads	[38]
CA (M_n_ 3.0 × 10^4^)	pure acetone	19 wt%	20; 10; 1.5	50–65 µm (transverse size)	flat/ribbon; fused junctions	[38]
cellulose (DP 210, 1140)	LiCl/DMAc + water coagulation	1–3 wt%	15–25; 10–20; 0.3–1.8	0.25–0.75 µm	uniform, amorphous	[39]
cellulose (cotton, wood pulp)	TFA	4–5 wt%	15; 15; N.R.	0.07–0.13 µm	fibers (pure cellulose)	[40]

DP: degree of polymerization; DS: degree of substitution; M_n_: number-average molar mass; M_w_: weight-average molar mass; MeOH: methanol; CHCl_3_: chloroform; LiCl: lithium chloride; TFA: trifluoroacetic acid; Conc.: concentration; Ref.: reference. Molar masses are expressed in g mol^−1^. ^a^ Concentrations are given in the units reported by each study. wt% denotes the mass of polymer per mass of solution (*w*/*w*); % (*w*/*v*) denotes grams of polymer per 100 mL of solvent (ref. [36] only). The two are not directly interconvertible without the solvent density, so values expressed in % (*w*/*v*) should not be read against the wt% values of the other rows. ^b^ Applied voltage (kV); needle-to-collector distance (cm); solution flow rate (mL/h), in that order and as reported in each study. *v*/*v*, volume ratio; w/w, mass ratio. Values originally given in inches (refs. [33,35]) or in mL/min (ref. [39]) have been converted. N.R., not reported. In ref. [33] the solution was fed as a pendent drop from a capillary tip without a pump, so no flow rate was reported; in ref. [35] a syringe pump was used but the feeding rate was not stated; in ref. [36] the solution was fed from a tilted syringe without a pump; in refs. [37,40] the flow rate is not given. Where no mass- or volume-ratio basis is indicated, the basis was not explicitly specified in the original study. This table summarizes outcomes from separate studies. Comparisons should account for polymer identity, molecular weight/DP, DS, concentration, additives, and spinning conditions. Unmatched entries indicate feasibility under the reported conditions, rather than a ranking of solvent performance; the comparison criteria are explained in Section 3.4.

**Table 4:** Representative alternative solvent systems for electrospinning of cellulose and its derivatives.

Substrate	Solvent System	Conc. ^a^	Conditions ^b^ (kV; cm; mL/h)	Fiber Diameter	Morphology	Ref.
CA (30,000)	Acetone/water; 15 wt% water	17 wt%	8–12; 6–12; 5–20	N.R.	Continuous fibers	[34]
CA (M_n_ 30,000)	Acetone/water 80:20 (*v*/*v*)	10 wt%	13; 10; 4	N.R.	Reduced beading	[43]
EC (22,800)	Ethanol/water 80:20 (*v*/*v*)	25 wt%	15; 10; 6	~3.8 µm	Continuous fibers	[43]
CA (30,000)	Acetic acid/water 70:30–95:5 (*w*/*w*)	17 wt%	25; 10; 3	160–1280 nm	Fibers; diameter varies with solvent ratio	[44]
CA (M_w_ 30,000; DS 2.45)	Acetic acid/water 2:1	20 wt%	17; 15; N.R.	140 ± 10 nm	Particles and fibers	[37]
CA (M_n_ 30,000)	Acetic acid/water 3:1	17 wt%	20; 10; 1.5	180–280 nm	Fibers with few beads	[38]
CMC (M_w_ 120,000; DS 0.72)	Water; CMC/PEO 1:1 (*w*/*w*)	4 + 4 wt%	35; 20; N.R.	~200–250 nm	Nanofibers; PEO subsequently extracted	[45]
HPMC (M_w_ 350,000)	Water/ethanol 1:1	2.86 wt%	35; 20; N.R.	128 nm	Homogeneous fiber sheet	[45]
MC (M_w_ 350,000)	Water/ethanol 1:1	2.86 wt%	40; 20; N.R.	N.R.	Nonuniform, partly fused fibers	[45]
Cellulose (DP 210)	NMMO/water 85:15 (*w*/*w*); propyl gallate 0.5–1 wt% of cellulose	9 wt%	15–25; 15; 0.6; nozzle 70 °C; water coagulation	~300 nm	Uniform fibers	[39]
Cellulose (DP 500)/PEG	NaOH/urea/water 7:12:81 (*w*/*w*/*w*); cellulose/PEG 8:2 (*w*/*w*)	3.8 wt% total	25; 15; N.R.; dissolution at −12 °C	~400 nm	Homogeneous blend fibers	[46]
Cellulose (194,400)	BmimCl/DMSO (15 g/0.05 g)	4 wt%	15; 15; N.R.; 100 °C; water coagulation	500–800 nm	Fibers	[47]
Cellulose (M_w_ 53,000)	EmimAc; or EmimAc/[C_10_mim]Cl 9:1 (mol/mol)	8 wt%	20; 12; 0.12; water coagulation	470 ± 110 nm; 120 ± 55 nm, respectively	Regenerated fibers	[48]
Bacterial cellulose (M_w_ 1,800,000)	BmimAc/DMSO or BmimAc/GVL, each 1:3 (*w*/*w*)	3 wt%	20–23; 9; 0.3; water coagulation	~0.5 µm	Fibers with either cosolvent	[27]
CA (50,000)	DMC/cyclopentanone 75:25 (*v*/*v*); TBAB 2.5 wt% of CA	12.5% (*w*/*v*)	15; 12.5; 0.5	286 ± 140 nm	Uniform fibers	[25]
CA	DMC/DMSO 50:50 (*w*/*w*)	18 wt%	15; 20; 150 ^c^	~0.5 µm	Smooth fibers	[49]
CA (M_n_ ~30,000)	Acetone/DMSO 77.5:22.5 (*v*/*v*)	14% (*w*/*v*)	16; 10; 0.9	317 ± 123 nm	Bead-free fibers	[50]
Cellulose pulp	DBU/DMSO + CO_2_; DBU 3 equivalents per anhydroglucose unit	4 wt%	29; 20; 0.12; dissolution under 5 bar CO_2_	~500 nm	Bead-free standing fibers	[51]

^a^ PEO, poly(ethylene oxide); HPMC, hydroxypropyl methylcellulose; MC, methylcellulose; DBU, 1,8-diazabicyclo[5.4.0]undec-7-ene. Selected formulations from the studies discussed in Section 3 are shown. Molecular weights are in g mol^−1^; values without M_n_ or M_w_ retain an unspecified averaging convention. Concentrations retain the units reported in the original studies; “total” denotes combined polymer content. ^b^ Conditions are applied voltage, needle-to-collector distance and solution flow rate, in that order. Essential heating, dissolution and coagulation conditions are included where relevant. Where no mass- or volume-ratio basis is indicated, the basis was not explicitly specified in the original study. The comparison criteria in Section 3.4 apply to polymer identity, molecular weight/DP, DS, concentration, additives, and processing conditions; unmatched entries indicate formulation-specific feasibility, not relative solvent performance. ^c^ Ref. [49] prints a flow rate of 2.5 mL/min, equivalent to 150 mL/h; the reported value is retained without correction.

## Data Availability

No new experimental datasets were generated. The literature sources supporting this review are cited in the article; sources of boiling-point data are provided in the Supplementary Materials.

## Supplementary Materials

The following supporting information can be downloaded at: https://www.mdpi.com/article/10.3390/polym18212765/s1. Supporting_Information.docx contains Table S1, documenting the sources and reporting status of boiling-point data used in Table 2.

## Abbreviations

BmimAc, 1-butyl-3-methylimidazolium acetate; BmimCl, 1-butyl-3-methylimidazolium chloride; CA, cellulose acetate; CMC, carboxymethyl cellulose; DESs, deep eutectic solvents; DBU, 1,8-diazabicyclo[5.4.0]undec-7-ene; DCM, dichloromethane; DMAc, N,N-dimethylacetamide; DMC, dimethyl carbonate; DMF, N,N-dimethylformamide; DMSO, dimethyl sulfoxide; EC, ethyl cellulose; EmimAc, 1-ethyl-3-methylimidazolium acetate; GVL, γ-valerolactone; HPMC, hydroxypropyl methylcellulose; HSPs, Hansen solubility parameters; IL, ionic liquid; ILs, ionic liquids; MC, methylcellulose; MeOH, methanol; NMMO, N-methylmorpholine N-oxide; PEG, poly(ethylene glycol); PEO, poly(ethylene oxide); PVA, poly(vinyl alcohol); TBAB, tetrabutylammonium bromide; TFA, trifluoroacetic acid; [C_10_mim]Cl, 1-decyl-3-methylimidazolium chloride; LiCl, lithium chloride; NaOH, sodium hydroxide; CHCl_3_, chloroform; CLP, Classification, Labelling and Packaging; REACH, Registration, Evaluation, Authorisation and Restriction of Chemicals; RED, relative energy difference; SDS, safety data sheet; MSDS, material safety data sheet; N.R., not reported; Mn, number-average molar mass; Mw, weight-average molar mass; DP, degree of polymerization; DS, degree of substitution; Flam. Liq., flammable liquid; Acute Tox., acute toxicity; Skin Corr., skin corrosion; Skin Irrit., skin irritation; Eye Irrit., eye irritation; Skin Sens., skin sensitization; Carc., carcinogenicity; Repr., reproductive toxicity; STOT SE/RE, specific target organ toxicity following single/repeated exposure; Aquatic Chronic, long-term aquatic hazard; H-statements, hazard statements; EUH, supplemental EU hazard statement; EU, European Union.
