# Supplementary material for: Toward Greener Solvent Systems for the Electrospinning of Cellulose and Its Derivatives
Source: Polymers (Basel). 2026 Nov 6;18(21):2765. doi: 10.3390/polym18212765 (PMC13765996; doi:10.3390/polym18212765)
Supplement: Supplementary file 1 [file polymers-18-02765-s001.zip › polymers-4680797-supplementary.pdf]

Supporting Information

Toward Greener Solvent Systems for the Electrospinning of Cellulose and Its Derivatives

Hoik Lee [1,2]

[1] Korea Institute of Industrial Technology (KITECH), Ansan 15588, Republic of Korea; hoik@kitech.re.kr

[2] Department of Chemical Engineering, College of Engineering, Chung-Ang University, Seoul, Republic of Korea

Contents

Table S1. Sources and reporting status of boiling-point data in Table 2.

Reference for Table S1.

Table S1. Sources and reporting status of the boiling-point data in Table 2.

Supplier safety data sheets (SDSs), material safety data sheets (MSDSs), and product specifications below document physical-property data for the identified products. CHEM21 boiling points are reproduced from the published guide [S1].

Substance	Source and product identification	SDS revision date	Boiling point or reporting status	Source
Acetone	Prat et al. [S1]; CHEM21 guide, Table 7	—	56 °C	Article
Methanol	Prat et al. [S1]; CHEM21 guide, Table 7	—	65 °C	Article
Acetic acid	Prat et al. [S1]; CHEM21 guide, Table 7	—	118 °C	Article
Chloroform	Prat et al. [S1]; CHEM21 guide, Table 7	—	61 °C	Article
Dichloromethane	Prat et al. [S1]; CHEM21 guide, Table 7	—	40 °C	Article
N,N-Dimethylformamide (DMF)	Prat et al. [S1]; CHEM21 guide, Table 7	—	153 °C	Article
N,N-Dimethylacetamide (DMAc)	Prat et al. [S1]; CHEM21 guide, Table 7	—	166 °C	Article
Formic acid	Prat et al. [S1]; CHEM21 guide, Table 7	—	101 °C	Article
Pyridine	Prat et al. [S1]; CHEM21 guide, Table 7	—	115 °C	Article
Trifluoroacetic acid (TFA)	Sigma-Aldrich; 299537; MSDS, Section 9	2004-04-04	72 °C at 760 mmHg	MSDS
Water	Prat et al. [S1]; CHEM21 guide, Table 7	—	100 °C	Article
Ethanol	Prat et al. [S1]; CHEM21 guide, Table 7	—	78 °C	Article
Dimethyl carbonate (DMC)	Prat et al. [S1]; CHEM21 guide, Table 8	—	90 °C	Article
Dimethyl sulfoxide (DMSO)	Prat et al. [S1]; CHEM21 guide, Table 7	—	189 °C	Article
Cyclopentanone	Sigma-Aldrich; C112402; product page	—	130–131 °C (literature value)	Product page
γ-Valerolactone (GVL)	Prat et al. [S1]; CHEM21 guide, Table 8	—	207 °C	Article
N-Methylmorpholine N-oxide (NMMO), monohydrate	Thermo Fisher Scientific; NMMO monohydrate, A15996; SDS, Section 9	2025-09-24	N.R. (No information available)	SDS
1-Ethyl-3-methylimidazolium acetate (EmimAc)	Tokyo Chemical Industry (TCI); E0883; SDS, Section 9	2025-11-11	N.R. (No data available)	SDS
1-Butyl-3-methylimidazolium acetate (BmimAc)	FUJIFILM Wako; 358-27292 / 356-27293; SDS, Section 9	2023-04-05	N.R. (No data available)	SDS
1-Butyl-3-methylimidazolium chloride (BmimCl)	TCI; B2194; European Union SDS, Section 9	2025-02-12	N.R. (No data available)	SDS

Substance	Source and product identification	SDS revision date	Boiling point or reporting status	Source
1-Decyl-3-methylimidazolium chloride ([C_{10}mim]Cl; additive)	TCI; D5351; United States SDS, Section 9	2024-11-07	N.R. (No data available)	SDS
Lithium chloride (LiCl)	Albemarle; lithium chloride, low sodium; product page	—	1382 °C	Product page
Sodium hydroxide (NaOH)	Fisher Chemical; S492065; product specification	—	1390 °C	Specification
Urea	Organisation for Economic Co-operation and Development (OECD); urea assessment; physical-property data	—	Decomposes before boiling	Assessment
Tetrabutylammonium bromide (TBAB)	Carl Roth; 6633; Great Britain SDS, Section 9	2024-09-18	N.R. (Not determined)	SDS
Poly(ethylene glycol) (PEG; high molecular weight)	Thermo Fisher Scientific; 18322; molecular weight 600,000–1,000,000; SDS, Section 9	2025-11-07	N.R. (No information available)	SDS
Poly(vinyl alcohol) (PVA)	Thermo Fisher Scientific; 41243; high molecular weight, 98–99% hydrolyzed; SDS, Section 9	2025-11-07	N.R. (No information available)	SDS
Sophorolipid biosurfactant (HoneySurf)	HoneySurf; product-specific SDS not located	—	Not verified	—

N.R. = no numerical boiling point reported in the cited SDS; the source wording is given in parentheses. N.R. does not establish that boiling is physically inapplicable. Decomposition before boiling is stated separately. “Not verified” identifies a product-specific value that could not be established.

Dates are those printed on the SDS or MSDS. A dash indicates a non-SDS source or the absence of a product-specific SDS. Online sources accessed September 22, 2026. The PEG and PVA SDSs describe representative grades and do not establish the exact grades used in every electrospinning study. The NMMO SDS refers specifically to its monohydrate. A product-specific HoneySurf SDS was not located.

Reference for Table S1

[S1] Prat, D.; Wells, A.; Hayler, J.; Sneddon, H.; McElroy, C. R.; Abou-Shehada, S.; Dunn, P. J. CHEM21 Selection Guide of Classical- and Less Classical-Solvents. Green Chem. 2016, 18, 288–296. https://doi.org/10.1039/C5GC01008J
